# Comprehensive risk factor-based nomogram for predicting one-year mortality in patients with sepsis-associated encephalopathy

**DOI:** 10.1038/s41598-024-74837-z

**Published:** 2024-10-14

**Authors:** Guangyong Jin, Menglu Zhou, Jiayi Chen, Buqing Ma, Jianrong Wang, Rui Ye, Chunxiao Fang, Wei Hu, Yanan Dai

**Affiliations:** 1 Department of Critical Care Medicine, Affiliated Hangzhou First People’s Hospital, School of Medicine, Westlake University, Hangzhou, Zhejiang Province People’s Republic of China; 2 Department of Critical Care Medicine, Hangzhou Geriatric Hospital, Hangzhou, Zhejiang Province People’s Republic of China; 3 Department of Neurology, Affiliated Hospital of Hangzhou Normal University, Hangzhou, Zhejiang Province People’s Republic of China

**Keywords:** Critical care, Encephalopathy, Malignant cancer, Nomogram, Sepsis, Data mining, Outcomes research, Brain injuries, Encephalopathy, Risk factors

## Abstract

Sepsis-associated encephalopathy (SAE) is a frequent and severe complication in septic patients, characterized by diffuse brain dysfunction resulting from systemic inflammation. Accurate prediction of long-term mortality in these patients is critical for improving clinical outcomes and guiding treatment strategies. We conducted a retrospective cohort study using the MIMIC IV database to identify adult patients diagnosed with SAE. Patients were randomly divided into a training set (70%) and a validation set (30%). Least absolute shrinkage and selection operator regression and multivariate logistic regression were employed to identify significant predictors of 1-year mortality, which were then used to develop a prognostic nomogram. The model’s discrimination, calibration, and clinical utility were assessed using the area under the receiver operating characteristic curve (AUC), calibration plots, and decision curve analysis, respectively. A total of 3,882 SAE patients were included in the analysis. The nomogram demonstrated strong predictive performance with AUCs of 0.881 (95% CI: 0.865, 0.896) in the training set and 0.859 (95% CI: 0.830, 0.888) in the validation set. Calibration plots indicated good agreement between predicted and observed 1-year mortality rates. The decision curve analysis showed that the nomogram provided greater net benefit across a range of threshold probabilities compared to traditional scoring systems such as Glasgow Coma Scale and Sequential Organ Failure Assessment. Our study presents a robust and clinically applicable nomogram for predicting 1-year mortality in SAE patients. This tool offers superior predictive performance compared to existing severity scoring systems and has significant potential to enhance clinical decision-making and patient management in critical care settings.

**Supplementary Information:**

The online version contains supplementary material available at 10.1038/s41598-024-74837-z.

Sepsis is defined as a life-threatening organ dysfunction caused by a dysregulated host response to infection^1^. The nervous system is often the first organ system to exhibit functional impairment due to sepsis, leading to sepsis-associated encephalopathy (SAE)^2^. SAE manifests as diffuse brain dysfunction stemming from the systemic inflammatory response to sepsis, with clinical presentations ranging from mild delirium to severe coma, and is associated with increased mortality and long-term physical, mental, and cognitive impairments^3–5^. 

The severity of SAE correlates with short-term mortality, with more severe consciousness disorders indicating a poorer prognosis^3^. Notably, even mild mental status changes are independently linked to an increased risk of short-term mortality^3^. Beyond short-term outcomes, acute sepsis-related neurological disorders are the organ dysfunction most closely associated with long-term mortality and are a key mediator of adverse long-term outcomes following sepsis^6^. Among sepsis survivors who experienced acute neurological dysfunction, the 1-year mortality rate is increased by an absolute 6% compared to those without such dysfunction^6^. Long-term prognosis is a crucial outcome, reflecting the overall burden of the disease. Accurate prediction of long-term mortality rates enhances doctor-patient communication and informs sustainable medical management decisions^7^.

As the number of sepsis survivors rises^5,8^, identifying risk factors associated with the long-term prognosis of SAE patients, particularly those that are conventional, readily available, and cost-effective, is vital for developing optimized treatment strategies and assessing patient outcomes. This study aims to identify predictive factors for 1-year mortality in SAE patients and to establish and validate a 1-year mortality prediction model in the form of a nomogram, thereby providing clinicians with a valuable tool for evaluating patient prognosis.

## Methods

### Database and study design

The study utilized the Medical Information Mart for Intensive Care IV (MIMIC IV, Version 2.1) database, which contains de-identified health data from over 40,000 patients admitted to the Beth Israel Deaconess Medical Center (BIDMC) in Boston, Massachusetts, from 2008 to 2019^9^. MIMIC IV includes comprehensive information on patient demographics, vital signs, laboratory test results, medications, procedures, and clinical outcomes, making it a valuable resource for conducting epidemiological and clinical research in critical care settings^7^.

The MIMIC IV database is de-identified in accordance with the Health Insurance Portability and Accountability Act, thereby safeguarding patient privacy. Consequently, its utilization for research does not necessitate additional patient consent. Researchers obtained access to the MIMIC IV data by completing the requisite data use agreement and the Collaborative Institutional Training Initiative Program’s course on “Data or Specimens Only Research.” The Institutional Review Board of BIDMC reviewed and approved the processes for patient data collection and the establishment of research resources, granting exemptions for informed consent and endorsing data-sharing protocols. Given the retrospective design of this study, and to uphold patient confidentiality while ensuring compliance with authorized use of the MIMIC IV database, no further ethical approval was required. All methods were performed in accordance with relevant guidelines and regulations.

In alignment with the Transparent Reporting of a Multivariable Prediction Model for Individual Prognosis or Diagnosis (TRIPOD) guidelines^10^, this study aimed to develop and validate a prognostic model for 1-year mortality in patients with SAE. The study involved screening adult patients with SAE from the MIMIC IV database, ensuring the exclusion of encephalopathy attributable to non-septic causes. Patients were randomly assigned to either a training set or a validation set. Least absolute shrinkage and selection operator (LASSO) regression and multivariate binary logistic regression analyses were conducted on the training set to identify significant risk factors for 1-year mortality. These identified risk factors were then utilized to construct a mortality prediction model, represented as a nomogram. The model’s performance was rigorously assessed by evaluating its discrimination, calibration, and clinical utility in both the training and validation cohorts.

### Patient selection

**Figure 1:**
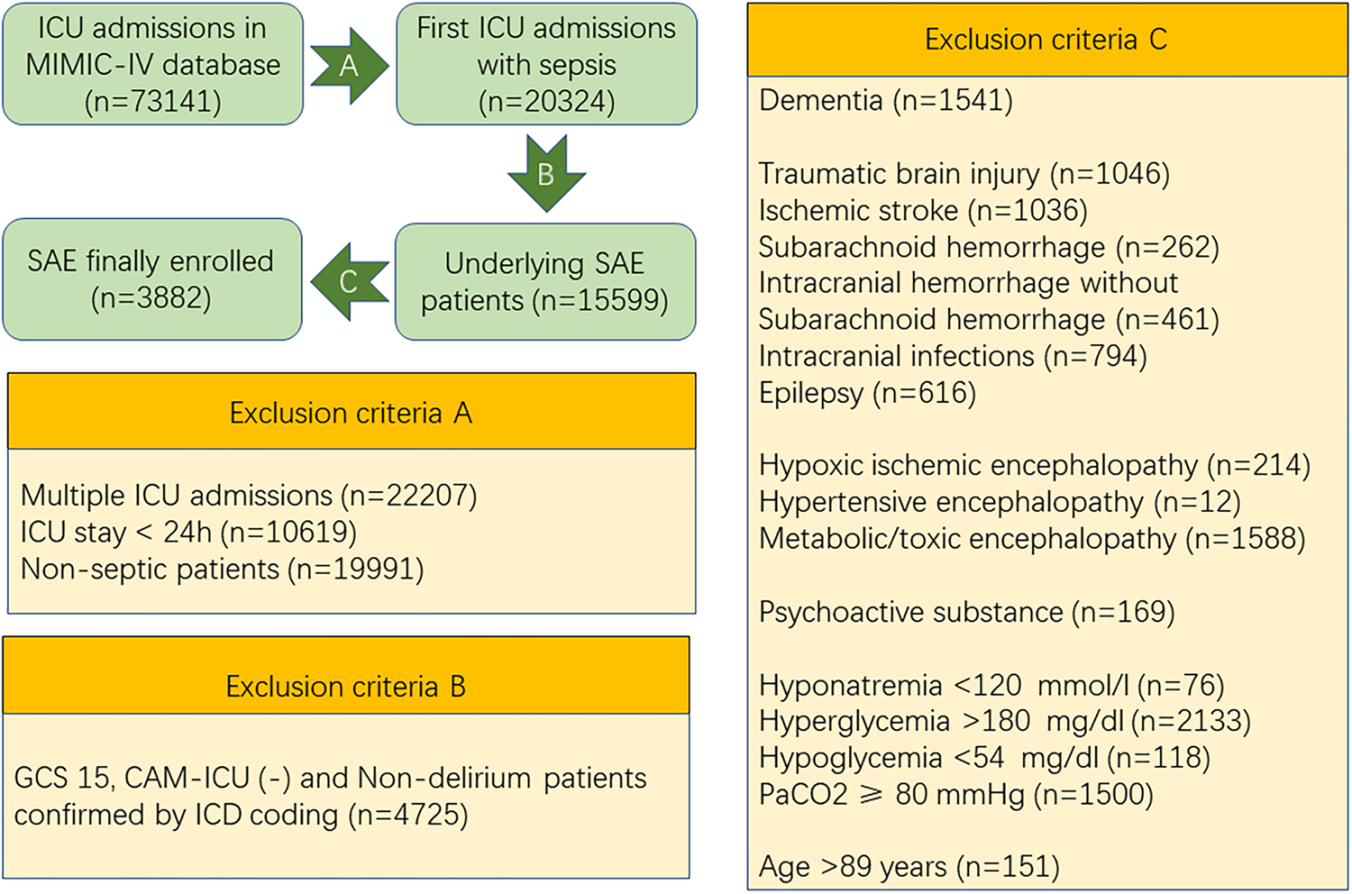
Selection Process and Exclusion Criteria for Research Subjects. CAM-ICU, Confusion Assessment Method for the ICU; GCS, Glasgow Coma Scale; MIMIC-IV, medical information mart for intensive care IV; ICD, International Classification of Diseases; ICU, intensive care unit; PaCO2, Partial pressure of CO2; SAE, Sepsis-Associated Encephalopathy.

The study cohort comprised patients with SAE who were admitted to the intensive care unit (ICU) for the first time. The inclusion criteria were as follows: (1) Sepsis 3.0 criteria: patients diagnosed with or suspected of infection and an increase of at least 2 points in the Sequential Organ Failure Assessment (SOFA) score; (2) Diagnosis of SAE on the first day of ICU admission, defined by a Glasgow Coma Scale (GCS) score of < 15, a positive Confusion Assessment Method for the ICU, or corresponding International Classification of Diseases codes (2930, 2931, F05). The exclusion criteria included: (1) Any record of subsequent ICU admissions; (2) ICU stays shorter than 24 h; (3) Non-septic conditions; (4) Patients with clear consciousness and no delirium symptoms; (5) Underlying dementia; (6) Primary brain injuries such as traumatic brain injury, ischemic stroke, hemorrhagic stroke, intracranial infection, or epilepsy; (7) Hypoxic-ischemic encephalopathy; (8) Hypertensive encephalopathy; (9) Metabolic or toxic encephalopathy; (10) Mental and behavioral disorders due to alcohol, drugs, or psychoactive substances; (11) Severe electrolyte and metabolic imbalances, including hyponatremia (< 120 mmol/L), hyperglycemia (> 180 mg/dL), hypoglycemia (< 54 mg/dL), and partial pressure of carbon dioxide (PaCO2) ≥ 80 mmHg; (12) Patients aged 89 years or older. A total of 3882 patients meeting the inclusion criteria were selected for analysis (Fig. 1).

### Data extraction and preprocessing

Research data were extracted from the MIMIC IV database, encompassing variables such as age, gender, weight, marital status, race, underlying comorbidities, and the Charlson Comorbidity Index (CCI). Clinical data from the first day of ICU admission included vital signs, key laboratory parameters, the use of invasive mechanical ventilation, renal replacement therapy (RRT), vasoactive medications, albumin administration, the GCS score, and other critical care scoring indices. Vasoactive medications comprised norepinephrine, epinephrine, dopamine, dobutamine, vasopressin, and milrinone. For variables with multiple measurements, the MIMIC concept was utilized to systematically extract the minimum, maximum, and average values where applicable. Time-related indicators were extracted to calculate ICU mortality, in-hospital mortality, 28-day mortality, 1-year mortality, length of hospital stay (LOS), and ICU LOS. The primary outcome measure was the 1-year mortality, determined by the survival status one year post ICU admission.

Data processing were conducted using Stata software (version 17.0, Stata Corporation LLC, College Station, USA). Outliers were identified through histogram analysis and addressed using the Winsorization technique via the Winsor2 command. Missing data were imputed using multiple imputation methods to ensure robust and accurate analysis.

### Study cohorts

Using R software (version 4.3.2, R Foundation for Statistical Computing, Vienna, Austria), the cohort of 3,882 patients with SAE was randomly divided into a training set (70%) and a validation set (30%) for the development and validation of predictive models, with the random seed set to 3684. The ‘gtsummary’ package was employed to perform univariate analyses on both the training and validation sets, assessing differences between the two groups and verifying the effectiveness of the randomization process. This approach ensures that both subsets are representative of the overall patient population, thereby supporting the robustness of the predictive models.

### Model development

Following the implementation of the ‘glmnet’ R package, regression analysis was performed. LASSO regression was utilized on the training set to discern significant risk factors for 1-year mortality. This technique facilitates feature selection by constraining less significant coefficients to zero, thereby enhancing model interpretability. Subsequently, the variables identified through LASSO regression were further analyzed using multivariate binary logistic regression to determine independent risk factors for 1-year mortality in patients with SAE. The ‘car’ R package was employed to calculate the variance inflation factor. Odds ratios (ORs) and 95% confidence intervals (CIs) were computed for each risk factor to quantify their association with 1-year mortality. This process culminated in the development of a robust mortality prediction model based on these identified risk factors.

### Nomogram construction and validation

Using the “rms” R package, a nomogram was developed from the final mortality prediction model based on logistic regression to visually represent the prediction model. This nomogram enables clinicians to estimate the probability of 1-year mortality for individual patients with SAE based on their specific clinical characteristics.

The discrimination, calibration, and clinical applicability of the mortality prediction model were assessed in both the training and validation sets. The “riskRegression” package was utilized to construct receiver operating characteristic (ROC) curves for the nomogram, SOFA, GCS score, Acute Physiology Score III (APS III), and Logistic Organ Dysfunction System (LODS), allowing evaluation of the model’s discriminative ability. Calibration curves were generated using the “val.prob” function to assess the agreement between predicted and observed outcomes. The “rmda” package facilitated decision curve analysis to evaluate clinical utility and ascertain the model’s net benefit across a range of threshold probabilities. Statistical significance was defined as a p-value < 0.05.

## Results

### Baseline characteristics

Comprehensive baseline characteristics of SAE patients in the entire cohort, as well as the training (*n* = 2751) and validation (*n* = 1131) subsets, are delineated in Table 1. Following extensive univariate analysis, no statistically significant disparities (*P* > 0.05) were observed across various demographic factors, comorbidities, disease severity indices, vital signs, essential laboratory parameters, or therapeutic interventions between the training and validation cohorts. Notably, the absence of significant discrepancies, particularly in 1-year mortality, underscores the rigorous adherence to scientific principles governing random allocation, thereby ensuring comparability across the cohorts. Patients with SAE were categorized into survival and death groups based on their 1-year survival outcomes. The supplementary materials provide a comprehensive comparison and analysis of the baseline characteristics of these two groups within both the training and validation sets.

**Table 1:** Baseline characteristics of patients with SAE in the overall cohort, Training Set, and Validation Set. *: Minimum recorded values of indicators during the first 24 h of ICU admission; **: Mean values of indicators during the first 24 h of ICU admission; ***: Maximum recorded values of indicators during the first 24 h of ICU admission. CCI, Charlson Comorbidity Index; ICU, Intensive Care Unit; IQR, Interquartile Range; GCS, Glasgow Coma Scale; APS III, Acute Physiology score III; SOFA, Sequential Organ Failure Assessment; LODS, logistic organ dysfunction system; MAP, Mean arterial pressure; PaCO2, partial pressure of CO2; SAE, Sepsis-Associated Encephalopathy. SAPS II, simplified Acute Physiology score II; OASIS, Oxford Acute Severity of Illness score; LOS, length of Stay.

Characteristics	All patients (*n* = 3882)	Training set (*n* = 2751)	Validation set (*n* = 1131)	*P*-value
Male, No. (%)	2,338 (60%)	1,671 (61%)	667 (59%)	0.307
Age, median (IQR)	67.26 (56.56, 76.43)	67.40 (56.43, 76.44)	66.90 (56.92, 76.39)	0.885
Height, median (IQR) (cm)	170.00 (163.00, 178.00)	170.00 (163.00, 178.00)	170.00 (161.50, 178.00)	0.285
Weight, median (IQR) (kg)	80.30 (68.10, 95.30)	81.00 (68.90, 95.55)	80.00 (67.10, 95.00)	0.067
**Race**,** No. (%)**				
White	2,705 (70%)	1,925 (70%)	780 (69%)	0.534
Hispanic	111 (2.9%)	74 (2.7%)	37 (3.3%)	0.323
Black	201 (5.2%)	147 (5.3%)	54 (4.8%)	0.467
Asian	100 (2.6%)	73 (2.7%)	27 (2.4%)	0.634
Other	765 (20%)	532 (19%)	233 (21%)	0.369
**Marital Status**,** No. (%)**				
Married	1,935 (50%)	1,384 (50%)	551 (49%)	0.368
Single	941 (24%)	673 (24%)	268 (24%)	0.612
Widowed	416 (11%)	283 (10%)	133 (12%)	0.178
Divorced	269 (6.9%)	184 (6.7%)	85 (7.5%)	0.357
Other	321 (8.3%)	227 (8.3%)	94 (8.3%)	0.951
**First Care Unit**,** No. (%)**				
Medical ICU	503 (13%)	362 (13%)	141 (12%)	0.560
Surgical ICU	389 (10%)	287 (10%)	102 (9.0%)	0.182
Medical ICU/Surgical ICU	447 (12%)	307 (11%)	140 (12%)	0.280
Neuro Surgical ICU	18 (0.5%)	13 (0.5%)	5 (0.4%)	0.899
Trauma Surgical ICU	401 (10%)	281 (10%)	120 (11%)	0.713
Other ICU	2,124 (55%)	1,501 (55%)	623 (55%)	0.767
**Underlying Diseases**,** No. (%)**				
Myocardial Infarction	701 (18%)	493 (18%)	208 (18%)	0.729
Congestive Heart Failure	1,025 (26%)	709 (26%)	316 (28%)	0.164
Chronic Pulmonary Disease	1,049 (27%)	729 (26%)	320 (28%)	0.253
Diabetes Without Chronic Complication	712 (18%)	502 (18%)	210 (19%)	0.815
Diabetes With Chronic Complication	201 (5.2%)	141 (5.1%)	60 (5.3%)	0.818
Rheumatic Disease	143 (3.7%)	94 (3.4%)	49 (4.3%)	0.169
Peptic Ulcer Disease	99 (2.6%)	78 (2.8%)	21 (1.9%)	0.079
Peripheral Vascular Disease	550 (14%)	393 (14%)	157 (14%)	0.743
Paraplegia	54 (1.4%)	40 (1.5%)	14 (1.2%)	0.601
Metastatic Solid Tumor	211 (5.4%)	146 (5.3%)	65 (5.7%)	0.583
Malignant Cancer	211 (5.4%)	146 (5.3%)	65 (5.7%)	0.583
**CCI**,** median (IQR)**	5.00 (3.00, 7.00)	5.00 (3.00, 7.00)	5.00 (3.00, 7.00)	0.342
**Vital Indicators**,** median (IQR)**				
Heart Rate (beats/min) *	70.00 (61.00, 80.00)	70.00 (61.00, 80.00)	70.00 (61.00, 79.00)	0.994
Heart Rate (beats/min) ***	101.00 (90.00, 115.00)	101.00 (90.00, 116.00)	100.00 (90.00, 115.00)	0.352
Heart Rate (beats/min) **	84.00 (76.00, 95.00)	84.00 (76.00, 95.00)	84.00 (76.50, 95.00)	0.903
MAP (mmHg) *	58.00 (52.00, 63.00)	58.00 (52.00, 63.00)	57.50 (52.00, 63.00)	0.620
MAP (mmHg) ***	98.00 (89.00, 109.00)	98.00 (89.00, 109.00)	98.00 (89.00, 109.00)	0.627
MAP (mmHg) **	74.54 (69.92, 79.65)	74.66 (70.10, 79.75)	74.28 (69.62, 79.50)	0.159
Respiratory Rate (breaths/min) *	12.00 (9.00, 14.00)	12.00 (9.00, 14.00)	12.00 (9.00, 14.00)	0.559
Respiratory Rate (breaths/min) ***	27.00 (23.00, 31.00)	27.00 (23.00, 31.00)	27.00 (24.00, 31.00)	0.152
Respiratory Rate (breaths/min) **	18.11 (16.19, 20.66)	18.06 (16.17, 20.65)	18.23 (16.21, 20.71)	0.292
Temperature (°C) *	36.33 (35.70, 36.61)	36.33 (35.67, 36.61)	36.33 (35.70, 36.61)	0.720
Temperature (°C) ***	37.40 (37.00, 37.94)	37.40 (37.00, 37.94)	37.40 (37.00, 37.94)	0.799
Temperature (°C) **	36.83 (36.56, 37.17)	36.83 (36.56, 37.17)	36.83 (36.57, 37.18)	0.587
First day Urine Output (mL)	1720.00 (1142.25, 2468.75)	1710.00 (1140.00, 2475.00)	1765.00 (1175.00, 2445.50)	0.449
**Laboratory Indicators**,** median (IQR)**				
Hemoglobin (g/L) *	93.00 (82.00, 107.00)	94.00 (82.00, 107.00)	93.00 (82.00, 106.00)	0.423
Hemoglobin (g/L) ***	112.00 (101.00, 126.00)	113.00 (101.00, 126.00)	112.00 (101.00, 126.00)	0.545
Platelets (K/uL) *	145.00 (107.00, 203.00)	145.00 (107.00, 200.00)	147.00 (108.00, 206.00)	0.129
Platelets (K/uL) ***	189.00 (145.00, 253.00)	188.00 (144.00, 249.00)	192.00 (148.00, 259.00)	0.051
White Blood Cells (K/uL) *	10.00 (7.30, 13.00)	9.90 (7.20, 13.00)	10.00 (7.50, 12.90)	0.547
White Blood Cells (K/uL) ***	14.70 (11.10, 19.00)	14.50 (11.00, 19.10)	15.10 (11.30, 18.95)	0.182
Anion Gap (mmol/L) *	11.00 (10.00, 13.00)	11.00 (10.00, 13.00)	12.00 (10.00, 13.00)	0.373
Anion Gap (mmol/L) ***	14.00 (12.00, 16.00)	14.00 (12.00, 17.00)	14.00 (12.00, 16.00)	0.839
Creatinine (µmmol/L) *	70.72 (61.88, 97.24)	70.72 (61.88, 97.24)	70.72 (61.88, 97.24)	0.851
Creatinine (µmmol/L) ***	88.40 (70.72, 123.76)	88.40 (70.72, 123.76)	88.40 (70.72, 123.76)	0.447
Glucose (mmol/L) *	6.06 (5.28, 6.94)	6.06 (5.28, 6.94)	6.06 (5.22, 6.94)	0.996
Glucose (mmol/L) ***	7.06 (6.06, 8.17)	7.06 (6.06, 8.17)	7.22 (6.11, 8.28)	0.077
Sodium (mEq/L) *	137.00 (135.00, 139.00)	137.00 (135.00, 139.00)	137.00 (135.00, 139.00)	0.386
Sodium (mEq/L) ***	140.00 (138.00, 142.00)	140.00 (138.00, 142.00)	140.00 (138.00, 142.00)	0.335
Potassium (mEq/L) *	4.00 (3.60, 4.30)	4.00 (3.60, 4.30)	4.00 (3.60, 4.30)	0.783
Potassium (mEq/L) ***	4.50 (4.20, 4.90)	4.50 (4.20, 4.90)	4.50 (4.20, 4.90)	0.196
Prothrombin Time (sec) *	13.40 (12.30, 14.90)	13.40 (12.30, 14.90)	13.40 (12.30, 14.80)	0.945
Prothrombin Time (sec) ***	15.30 (13.60, 17.50)	15.30 (13.60, 17.50)	15.30 (13.60, 17.50)	0.982
Partial Thromboplastin Time (sec) *	28.90 (26.20, 33.00)	28.80 (26.20, 33.00)	29.00 (26.20, 33.25)	0.473
Partial Thromboplastin Time (sec) ***	33.70 (29.10, 43.70)	33.70 (29.00, 43.69)	33.80 (29.35, 44.00)	0.481
Lactate (mmol/L) *	1.30 (0.92, 1.70)	1.30 (0.93, 1.70)	1.30 (0.91, 1.70)	0.705
Lactate (mmol/L) ***	2.20 (1.50, 3.16)	2.20 (1.50, 3.11)	2.20 (1.50, 3.20)	0.204
PH *	7.32 (7.27, 7.37)	7.32 (7.27, 7.37)	7.33 (7.27, 7.37)	0.992
PH ***	7.43 (7.39, 7.47)	7.43 (7.39, 7.47)	7.43 (7.39, 7.47)	0.384
PaCO2 (mmHg) *	35.00 (31.00, 39.00)	35.00 (32.00, 39.00)	35.00 (31.00, 39.00)	0.680
PaCO2 (mmHg) ***	46.00 (41.00, 52.00)	46.00 (41.00, 52.00)	46.00 (41.00, 52.00)	0.802
**Intervention Measures**,** No. (%)**				
Vasoactive Agent Use	2,420 (62%)	1,695 (62%)	725 (64%)	0.146
Albumin Use	95 (2.4%)	70 (2.5%)	25 (2.2%)	0.540
Renal Replacement Therapy	126 (3.2%)	96 (3.5%)	30 (2.7%)	0.181
Invasive Mechanical Ventilation	2,380 (61%)	1,684 (61%)	696 (62%)	0.850
Supplemental Oxygen Therapy	2,337 (60%)	1,667 (61%)	670 (59%)	0.433
**Disease severity Score**,** median (IQR)**				
First day GCS *	14.00 (9.00, 14.00)	14.00 (9.00, 14.00)	13.00 (8.00, 14.00)	0.162
First day SOFA	6.00 (4.00, 9.00)	6.00 (4.00, 9.00)	6.00 (4.00, 9.00)	0.173
First day APS III	46.00 (33.00, 69.00)	46.00 (32.00, 69.00)	46.00 (33.00, 70.00)	0.591
First day SAPS II	38.00 (29.00, 48.00)	37.00 (29.00, 48.00)	38.00 (30.00, 49.00)	0.112
First day OASIS	35.00 (29.00, 41.00)	35.00 (29.00, 41.00)	35.00 (29.00, 41.00)	0.562
First day LODS	5.00 (3.00, 8.00)	5.00 (3.00, 8.00)	5.00 (3.00, 8.00)	0.297
**Outcomes**				
1-year Mortality (%)	786 (20%)	557 (20%)	229 (20%)	> 0.999
ICU Mortality (%)	320 (8.2%)	238 (8.7%)	82 (7.3%)	0.149
Hospital Mortality (%)	385 (9.9%)	279 (10%)	106 (9.4%)	0.466
ICU LOS (days)	3.16 (1.59, 6.13)	3.13 (1.54, 6.04)	3.23 (1.77, 6.24)	0.170
Hospital LOS (days)	8.34 (5.42, 14.15)	8.26 (5.41, 13.83)	8.61 (5.42, 15.26)	0.108

### Development of the nomogram

**Figure 2:**
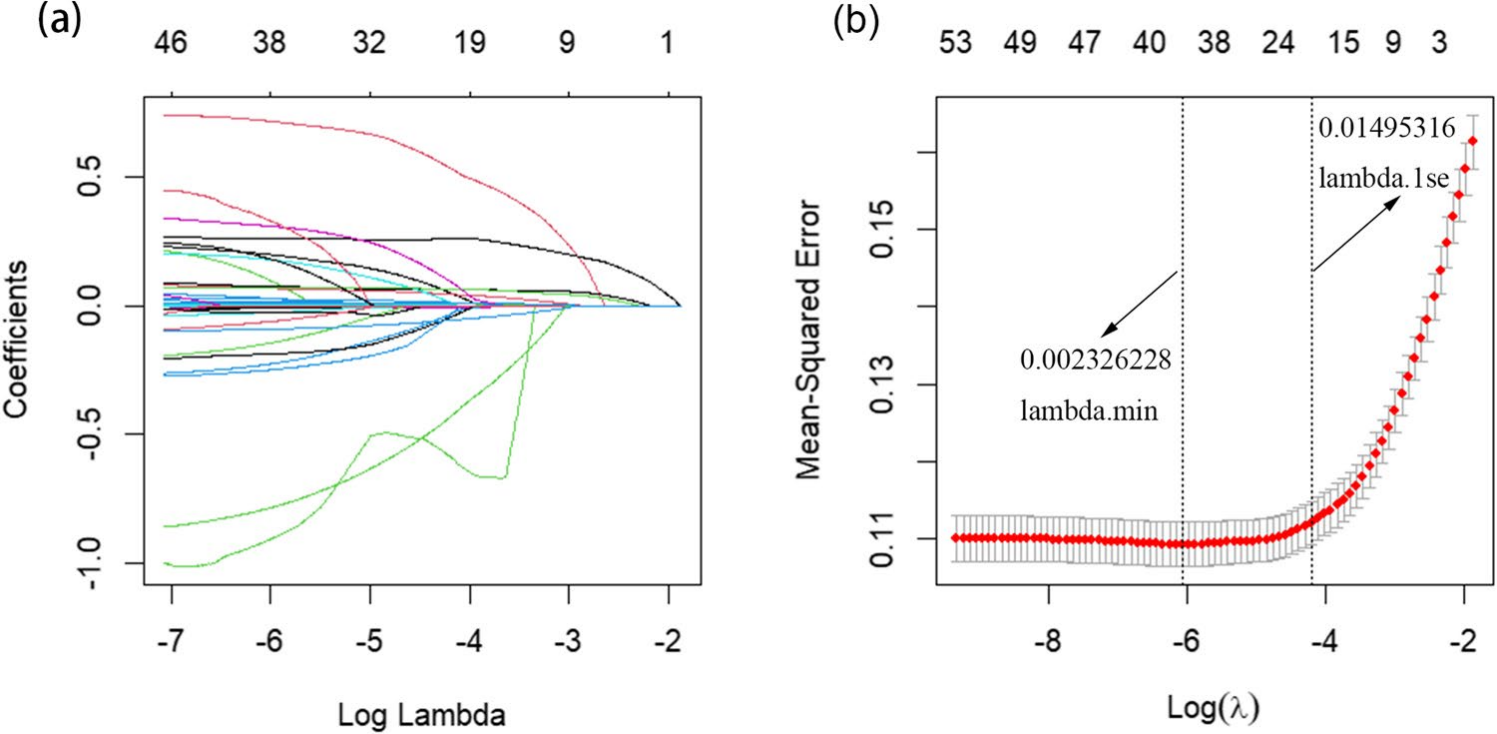
Feature Selection Process Using LASSO Regression and Tenfold Cross-Validation. (**a**) The graph illustrates the relationship between the coefficients of clinical features and the lambda values in LASSO regression. (**b**) The graph presents the tenfold cross-validation curve for LASSO regression, which aids in model selection. This figure provides a comprehensive view of the feature selection process and the criteria used for determining the optimal model parameters. LASSO, least absolute shrinkage and selection operator regression; λ, lambda.

**Figure 3:**
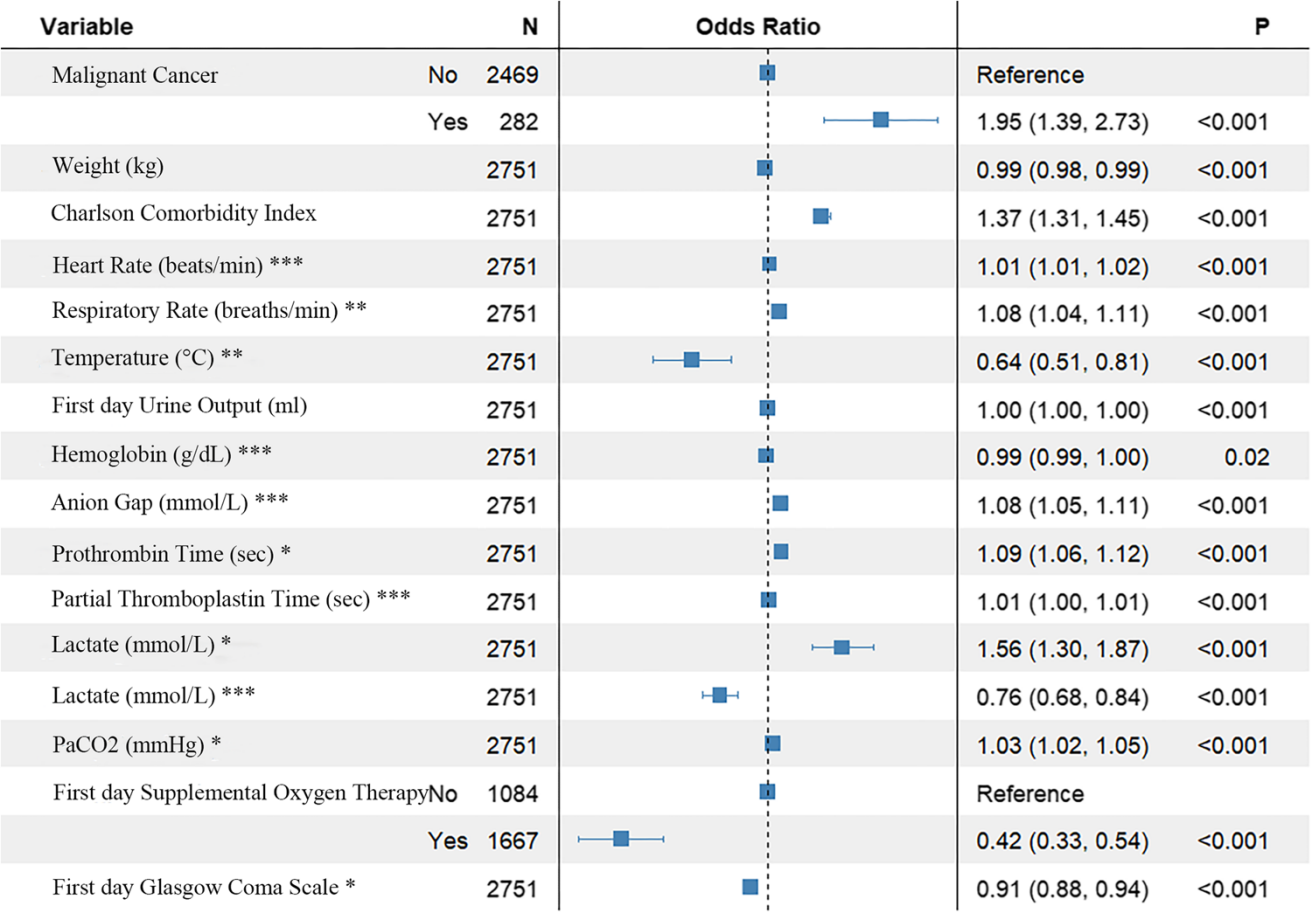
Multivariate Binary Logistic Regression Analysis for Predicting 1-Year Mortality in SAE Patients. This forest plot displays the results of the multivariate binary logistic regression analysis, identifying independent predictors of 1-year mortality among SAE patients in the training set. Each predictor is represented with its corresponding odds ratio and 95% confidence interval, providing a clear visualization of the significant factors contributing to the 1-year mortality risk. PaCO2, Partial pressure of CO2; SAE, Sepsis-Associated Encephalopathy. *: Minimum recorded values of indicators during the first 24 h of ICU admission; **: Mean values of indicators during the first 24 h of ICU admission; ***: Maximum recorded values of indicators during the first 24 h of ICU admission.

**Figure 4:**
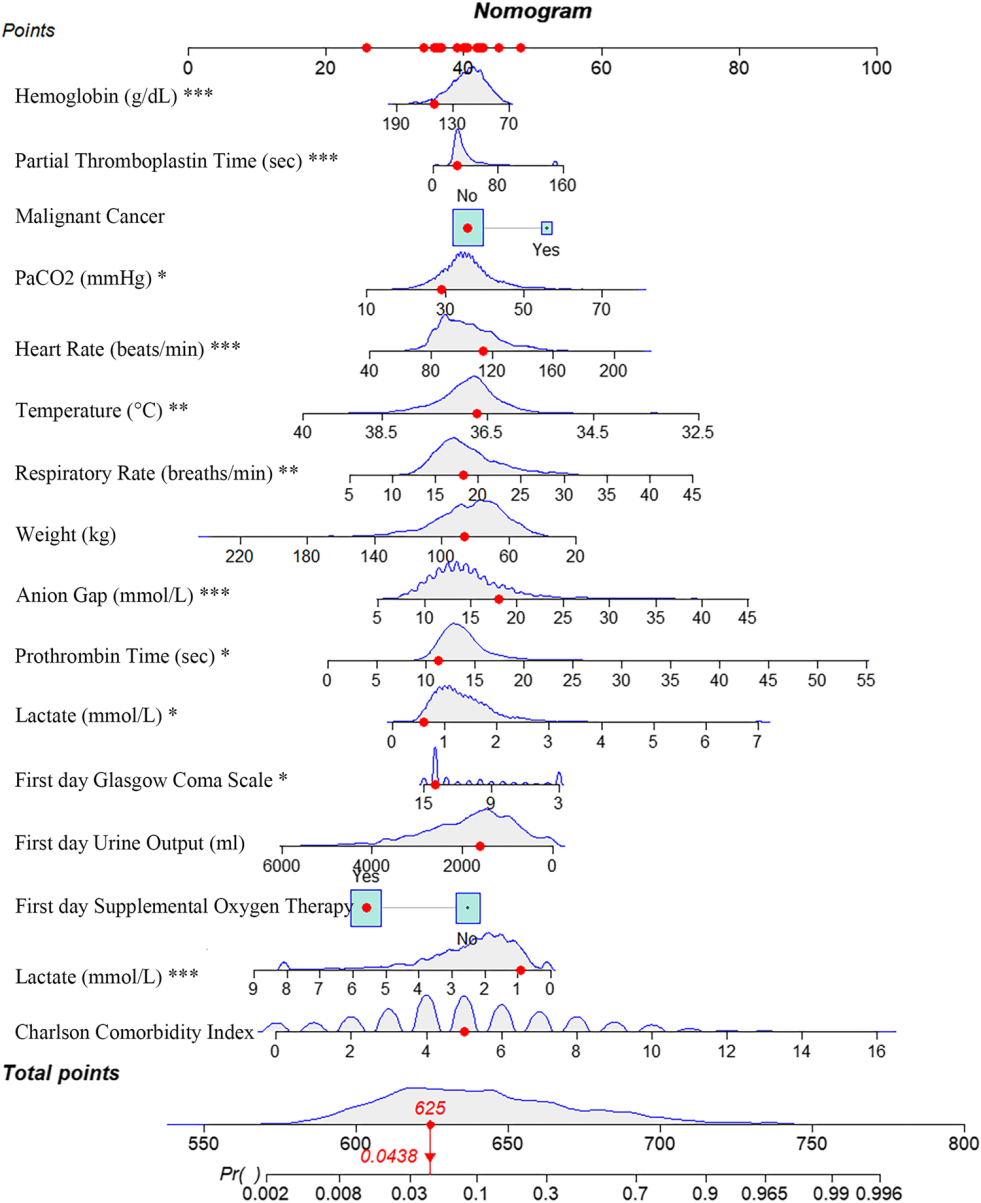
Nomogram for Predicting 1-Year Mortality in Patients with SAE. The nomogram operates by assigning points to specific variables based on their respective values. The total points are then summed and the corresponding 1-year mortality rate is determined by referencing the total point axis. The red dots in the figure represent a specific case of an SAE patient with the following characteristics: no history of malignant tumors, a Charlson Comorbidity Index score of 5, weight of 86.2 kg, maximum heart rate of 114 beats per minute, average respiration rate of 18 breaths per minute, average body temperature of 36.7 degrees Celsius, first day urine output of 1621 mL, maximum hemoglobin value of 150 g/dL, anion gap of 18 mmol/L, maximum partial thromboplastin time of 29.2 s, minimum prothrombin time of 11.3 s, maximum lactate value of 0.9 mmol/L, minimum lactate value of 0.6 mmol/L, PaCO2 of 29 mmHg, and minimum Glasgow Coma Scale score of 14. The patient also received treatment with supplemental oxygen. The total score for this patient was calculated to be 625, corresponding to a 1-year mortality rate of 4.38%. PaCO2, Partial pressure of CO2; SAE, Sepsis-Associated Encephalopathy. *: Minimum recorded values of indicators during the first 24 h of ICU admission; **: Mean values of indicators during the first 24 h of ICU admission; ***: Maximum recorded values of indicators during the first 24 h of ICU admission.

To identify predictors of 1-year mortality in patients with SAE, we evaluated numerous clinical variables. Using LASSO regression combined with 10-fold cross-validation, we initially screened for potential predictive factors (Fig. 2a and b). These preliminary factors were then subjected to binary multivariate regression analysis, resulting in the identification of 16 factors that were significantly associated with 1-year mortality in SAE patients (*P* < 0.05) (Fig. 3). Based on these predictive factors, a prognostic nomogram for 1-year mortality in SAE patients was subsequently developed (Fig. 4).

### Nomogram discrimination

**Figure 5:**
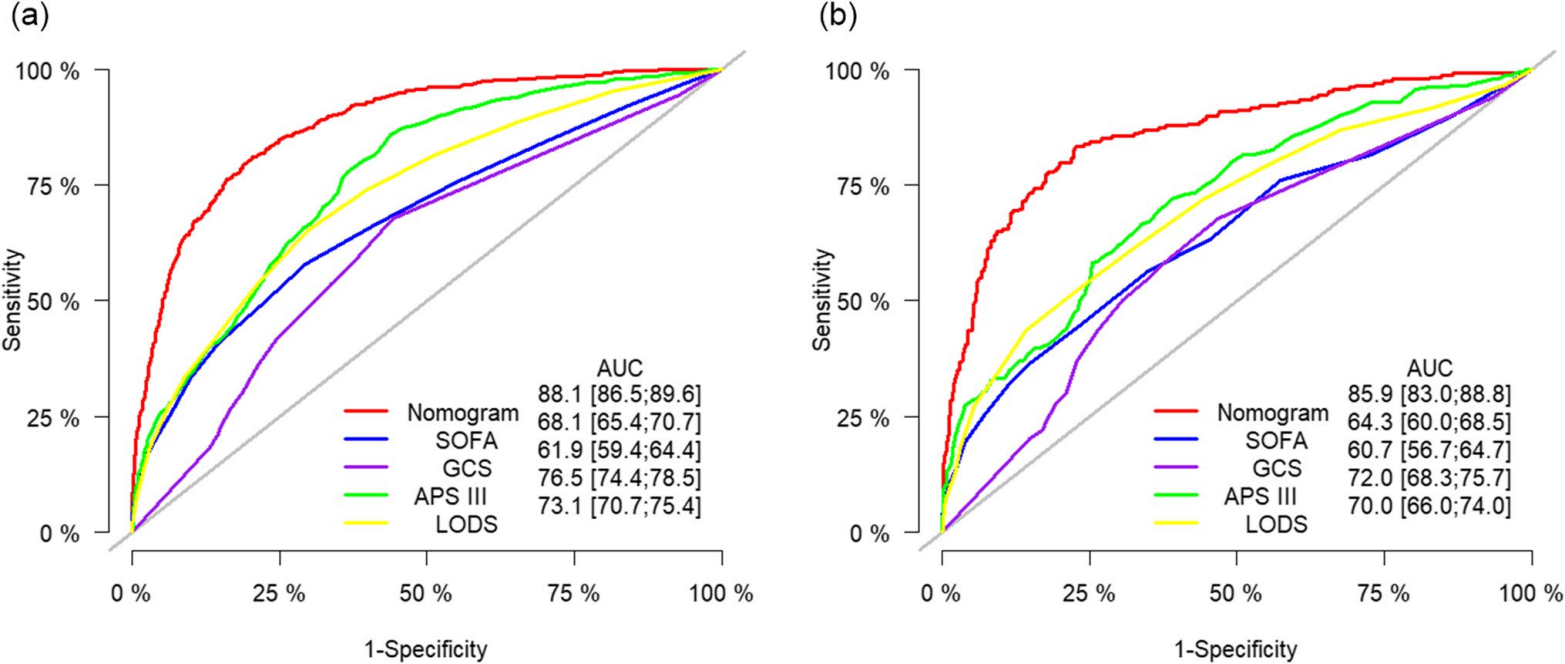
Comparison of Model Accuracy in Predicting 1-Year Mortality in SAE Patients. The graph compares the accuracy of different models in predicting the 1-year mortality rate of patients with sepsis-associated encephalopathy (SAE) in the training set (**a**) and validation set (**b**). The established nomogram demonstrated the highest area under the receiver operating characteristic curve in both datasets, indicating superior predictive performance compared to other models. APS III, Acute Physiology Score III; AUC, area under the receiver operating characteristic curve; GCS, Glasgow Coma Scale; LODS, Logistic Organ Dysfunction System; SAE, Sepsis-Associated Encephalopathy; SOFA, Sequential Organ Failure Assessment.

The developed prognostic nomogram demonstrated robust discrimination performance, with an area under the receiver operating characteristic (ROC) curve of 0.881 (95% CI, 0.865 to 0.896) in the training set and 0.859 (95% CI, 0.830 to 0.888) in the validation set (Fig. 5). These results indicate a high level of accuracy in predicting 1-year mortality among patients with SAE. Importantly, the discrimination performance of the constructed nomogram exceeded that of the GCS score and other commonly utilized disease severity scoring systems, underscoring its superior prognostic utility (Fig. 5). This enhanced predictive capability can provide clinicians with a more reliable tool for risk stratification and individualized patient management, ultimately may improving clinical outcomes for patients with SAE.

### Nomogram calibration

**Figure 6:**
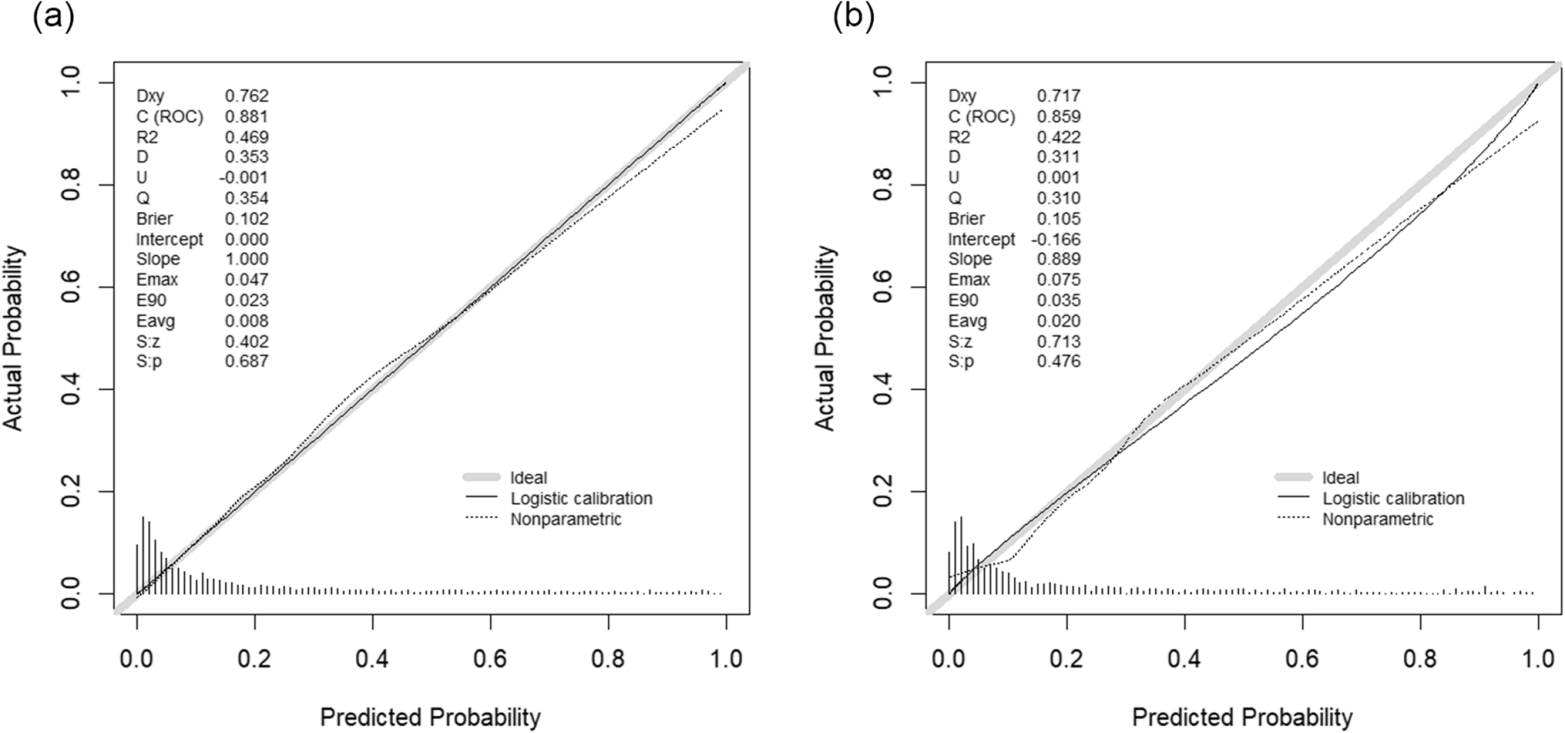
Calibration Curve of the Established Nomogram. The graph displays the calibration curves for the established nomogram, illustrating the agreement between predicted and observed 1-year mortality rates in the training set (**a**) and validation set (**b**). The curves demonstrate good consistency, indicating the nomogram’s reliable predictive performance in both datasets.

Calibration curves were drawn for the nomograms in both the training and validation sets to evaluate the accuracy of the prognostic nomogram. The analysis revealed a significant alignment between the predicted mortality rate and the actual 1-year mortality rate, underscoring the robust calibration performance of the model across both datasets (Fig. 6a, b). This consistency highlights the reliability of the nomogram in providing accurate mortality predictions for patients with SAE, thereby enhancing its clinical applicability for risk assessment and patient management.

### Nomogram clinical utility

**Figure 7:**
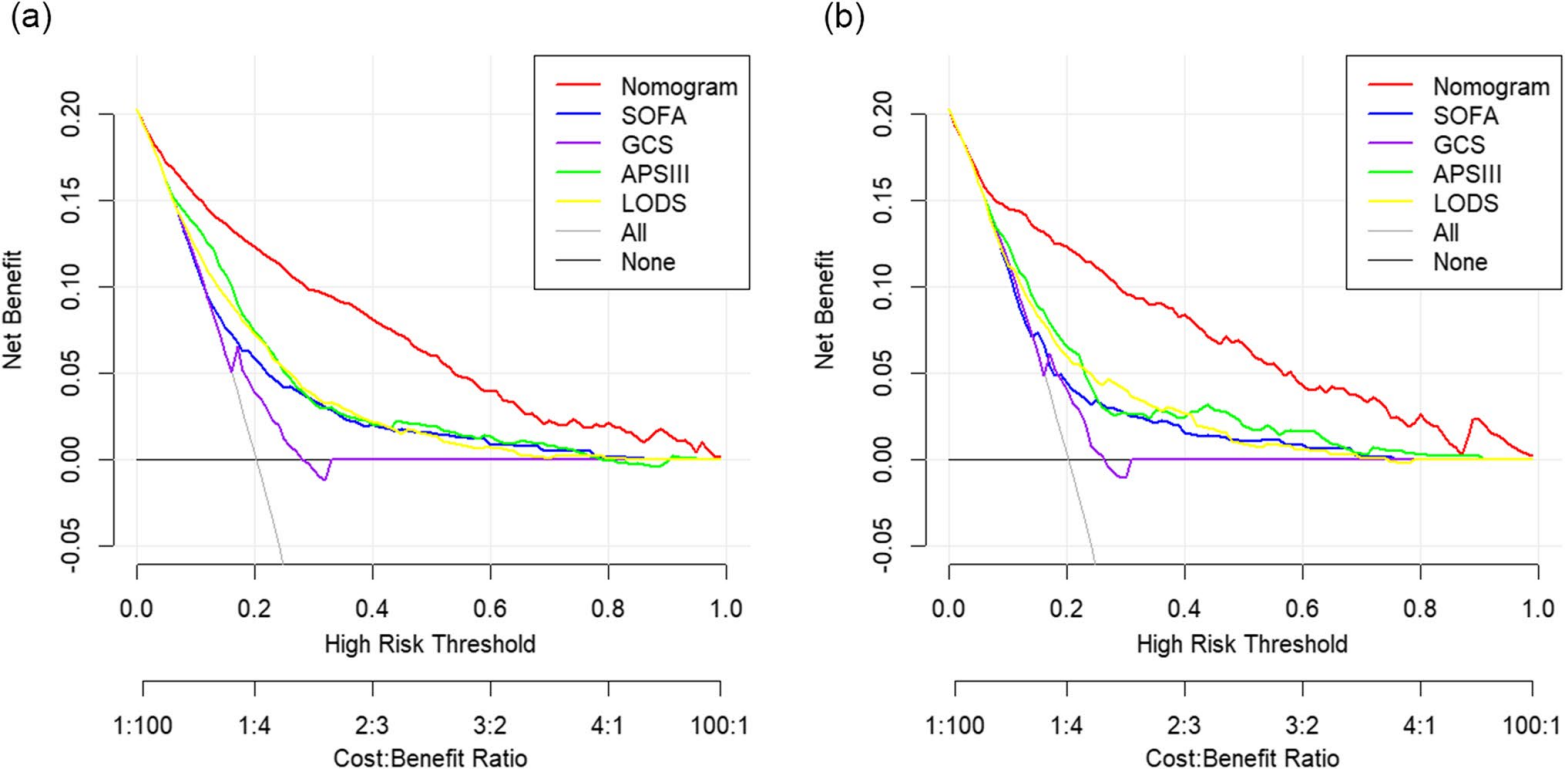
Decision Curve Analysis (DCA) for Predicting 1-Year Mortality in SAE Patients. The graph presents the DCA for various models predicting 1-year mortality in patients with SAE in the training set (**a**) and validation set (**b**). The curves assess the clinical utility of the models. The established nomogram demonstrates significantly higher clinical utility compared to other models in both datasets, indicating its superior performance in practical application. APS III, Acute Physiology Score III; AUC, area under the receiver operating characteristic curve; GCS, Glasgow Coma Scale; LODS, Logistic Organ Dysfunction System; SAE, Sepsis-Associated Encephalopathy; SOFA, Sequential Organ Failure Assessment.

Decision curve analysis (DCA) was employed to assess the clinical utility of the developed nomogram. Our nomogram demonstrated superior clinical utility compared to both the GCS score and other commonly used disease severity scoring systems (Fig. 7). This analysis underscores the enhanced practicality of our nomogram in clinical settings, providing clinicians with a more effective tool for prognostic assessment and patient management.

## Discussion

In this study, we developed and validated a prognostic nomogram to predict 1-year mortality in patients with SAE using a comprehensive set of clinical variables. Our findings demonstrate that the nomogram exhibits robust discriminative ability and calibration performance, surpassing traditional scoring systems such as the GCS score and other common disease severity indices. By integrating easily obtainable clinical parameters, our model provides clinicians with a practical and reliable tool for risk stratification in SAE, facilitating more informed decision-making and personalized patient management. These results underscore the importance of incorporating multifaceted clinical data to enhance prognostic accuracy and improve outcomes in critically ill patients with sepsis.

Our research has made substantial advancements compared to previous studies on prognostic models for SAE. Earlier studies primarily concentrated on short-term outcomes and, although useful for immediate clinical decision-making, often lacked the ability to predict long-term outcomes, particularly for SAE patients^11–15^. For instance, using the MIMIC III database, a user-friendly nomogram was created to predict 30-day mortality risk in SAE patients, with an AUC of 0.763 [0.736–0.791]^11^, indicating moderate predictive performance. Another study developed a nomogram based on clinical data to predict in-hospital mortality in SAE patients^12^. However, this model included the Simplified Acute Physiology Score II (SAPS II) score as a predictive variable, necessitating its prior completion, thereby increasing the model’s complexity and limiting its practical application.

Moreover, sophisticated machine learning methods have been employed to construct mortality prediction models, effectively predicting the 30-day mortality rate or ICU mortality rate in SAE patients^13,14^. Among these models, the APS III score emerged as a significant predictor, yet its necessity for model use adds complexity, hindering clinical adoption. A notable effort involved a stacking ensemble model that achieved a high AUC (0.807) in the test set and 0.671 in external validation for predicting ICU mortality risk in SAE patients using common clinical variables^15^.

In contrast, our nomogram, based on logistic regression, leverages common clinical features as predictive factors, making it both interpretable and straightforward. This simplicity enhances its clinical applicability and ease of use. By focusing on easily obtainable clinical features and ensuring transparency in how these factors influence mortality, our model offers a significant improvement in both predictive performance and practical implementation, facilitating broader clinical adoption.

In our predictive model, key risk factors for 1-year mortality in SAE patients include a history of malignancy, higher CCI scores, elevated minimum lactate levels, lower mean body temperature, and decreased maximum lactate levels. Conversely, SAE patients receiving oxygen supplementation exhibited lower 1-year mortality rates. Cancer patients are more susceptible to sepsis than the general population, with sepsis being a leading cause of ICU admissions among these individuals^16^. Compared to non-cancer sepsis patients, those with cancer have a significantly higher risk of late mortality (OR = 2.46, 95% CI: 1.42–4.25, I²=99%)^17^. Cancer patients undergo complex immune alterations, with treatments often inducing local or systemic inflammation as a result of tissue damage and the death of cancer cells^18^. Both chronic host state dysregulation due to cancer and acute host response dysregulation due to sepsis mediate mortality in sepsis patients with pre-existing malignant cancer^16^.

The CCI is a well-established predictor of outcomes in sepsis^19^, with comorbidities being a significant determinant of infection-related in-hospital mortality^20^. Accumulation of comorbid conditions is closely linked to increased severity of acute organ dysfunction, underscoring the critical role of comorbidities in the clinical course and prognosis of septic patients^21^. Consequently, the CCI serves as a valuable tool in stratifying risk and guiding clinical decision-making in patients with sepsis, underscoring the necessity of comprehensive comorbidity assessment in improving prognostication and individualized patient care.

Body temperature is inversely correlated with the prognosis of patients with sepsis-associated encephalopathy (SAE), aligning with previous research findings. A systematic review of 42 studies reported mortality rates of 22.2% for septic patients with a fever > 38 °C, 31.2% for normothermic patients, and 47.3% for hypothermic patients (< 36.0 °C)^22^. Fever appears to enhance the innate immune response, and many bacteria exhibit reduced replication at higher temperatures^23^. Conversely, hypothermia is common in sepsis and is associated with increased mortality^24^. Therefore, interventions aimed at warming patients with hypothermic sepsis may improve prognosis.

The measurement of serum lactate levels is a critical component in the clinical management of critically ill patients, particularly those with sepsis or septic shock^1^. Various metabolic changes in sepsis can elevate blood lactate levels, such as increased glycolysis, heightened Na-K pump activity stimulated by catecholamines, alterations in pyruvate dehydrogenase activity, and decreased lactate clearance due to impaired liver perfusion^25^. Elevated lactate levels are recognized as an independent risk factor for mortality in sepsis patients^26^, whereas lower lactate concentrations are associated with better outcomes^25^. Lactate clearance, defined by the change in lactate levels between two time points, is an efficient and cost-effective parameter that holds promise as a target for quantitative recovery^27^. Early lactate clearance-guided therapy has been linked to reduced mortality in sepsis^27^. Our study indicates that lower maximum lactate levels are associated with an increased risk of long-term mortality. This finding may initially seem counterintuitive, as elevated lactate levels are typically associated with worse outcomes. However, the interpretation of lactate levels must consider the overall clinical context and trends rather than isolated values. In this study, the association between lower maximum lactate levels and higher minimum lactate levels as indicators of poor prognosis in SAE patients indeed suggests a complex relationship. Specifically, when both lower maximum and higher minimum lactate levels are observed, it implies that the peak lactate level on the first day of ICU admission is relatively close to the trough, which may indicate suboptimal lactate clearance. This pattern could be indicative of inadequate metabolic recovery or persistent underlying issues, even if initial improvements are apparent. Thus, in the presence of other indicators of poor clinical progression, lower maximum lactate levels might reflect more complex clinical conditions that contribute to increased mortality risk. Unfortunately, due to the variability in timing for lactate remeasurement among patients and the absence of standardized protocols, precise lactate clearance rate data are not available in the MIMIC database. This limitation prevents us from obtaining standard lactate clearance rates and further elucidating their direct relationship with SAE mortality. We acknowledge this gap and agree that future research is needed to investigate the exact relationship between standard lactate clearance rates and SAE mortality. Such studies could provide valuable insights into the role of lactate dynamics in predicting outcomes and guide more effective management strategies for SAE patients.

The clinical implications of this study are substantial. The developed nomogram, based on readily available clinical variables, provides a practical and reliable tool for predicting 1-year mortality in patients with SAE. By incorporating this predictive model into clinical practice, healthcare providers can more accurately stratify patients based on their risk, facilitating personalized treatment strategies and informed decision-making. This model aids in identifying high-risk patients who may benefit from intensified monitoring and therapeutic interventions, thereby potentially improving clinical outcomes. Furthermore, the nomogram’s superior performance compared to traditional scoring systems underscores its value in enhancing prognostic accuracy. The use of decision curve analysis further emphasizes the model’s clinical utility, demonstrating its ability to offer significant net benefits across a range of threshold probabilities. Ultimately, the adoption of this predictive tool in clinical settings could lead to improved resource allocation, better patient management, and enhanced communication between clinicians and patients regarding prognosis and care plans.

This study offers several advantages. Firstly, the use of the MIMIC IV database, which includes a large and diverse patient cohort, enhances the generalizability and robustness of our research findings. The combination of LASSO regression and multivariate logistic regression ensures rigorous selection of predictive factors, thereby ensuring the correlation and accuracy of identified risk factors. Moreover, the development of nomograms incorporates a range of readily available clinical variables, making them practical tools for use across various clinical settings. The validation of the nomograms in both the training and validation cohorts underscores their reliability and robustness. Additionally, the inclusion of decision curve analysis to assess clinical utility provides valuable insights into the practical benefits of nomograms, highlighting their superior performance in predicting 1-year mortality rates in patients with sepsis-associated encephalopathy compared to traditional scoring systems. Therefore, this study not only introduces a novel predictive model but also establishes a benchmark for future research aimed at enhancing prognostic tools for critically ill patients.

Several limitations should be noted in this study. Firstly, the retrospective nature of our analysis may introduce inherent biases, including selection and information biases, potentially affecting the generalizability of our findings. Prospective studies are warranted to further validate the established nomogram. Additionally, while the comprehensive use of the MIMIC IV database provides robust data, its restriction to a single healthcare system may limit the applicability of our results to broader populations and settings. Therefore, we plan to conduct external validation of our prediction model in diverse settings. Furthermore, although the nomogram demonstrates strong predictive performance, it relies on variables available within the database. During variable selection, we carefully considered factors such as the feasibility of data collection, accessibility, and economic considerations. Finally, while our study included a wide array of clinical variables, the MIMIC-IV database has inherent limitations, including the absence of certain potential predictive factors such as some biomarkers, genetic data, electroencephalography, and cranial imaging examinations. These limitations underscore the need for further research to explore these additional factors and enhance our understanding of SAE. Future prospective studies should address these limitations and validate our findings across various medical contexts and patient populations.

## Conclusion

In conclusion, this study successfully developed and validated a robust nomogram for predicting 1-year mortality in patients with SAE. Utilizing a large and diverse cohort from the MIMIC IV database, our model demonstrated superior predictive performance compared to traditional scoring systems, underscoring its potential utility in clinical practice. The inclusion of easily obtainable clinical variables ensures the model’s practicality and accessibility, making it a valuable tool for risk stratification and personalized patient management. Furthermore, the decision curve analysis highlighted the clinical relevance and net benefit of the nomogram, affirming its potential to enhance prognostic accuracy and inform therapeutic decision-making. Future studies should focus on external validation and the incorporation of additional predictive factors to further refine and validate the model.

## Supplementary Information

Supplementary Material 1.

## Data Availability

The anonymised data collected are available as open data via the MIMIC data repository: https://physionet.org/content/mimiciv/2.1.

## Abbreviations

APS III: Acute Physiology Score III
AUC: area under the receiver operating characteristic curve
BIDMC: Beth Israel Deaconess Medical Center
CCI: Charlson Comorbidity Index
CI: confidence interval
DCA: decision curve analysis
GCS: Glasgow Coma Scale
ICU: Intensive Care Unit
LASSO: Least Absolute Shrinkage and Selection Operator regression
LODS: Logistic Organ Dysfunction System
LOS: length of stay
MIMIC-IV: Medical Information Mart for Intensive Care IV
OR: odd ratio
ROC: receiver operating characteristic
RRT: Renal Replacement Therapy
SAE: Sepsis-Associated Encephalopathy
SAPS II: Simplified Acute Physiology Score II
SOFA: Sequential Organ Failure Assessment
TRIPOD: Transparent Reporting of a multivariable prediction model for Individual Prognosis or Diagnosis
