# Supplementary material for: Comprehensive risk factor-based nomogram for predicting one-year mortality in patients with sepsis-associated encephalopathy
Source: Sci Rep. 2024 Oct 14;14:23979. doi: 10.1038/s41598-024-74837-z (PMC11473772; doi:10.1038/s41598-024-74837-z)
Supplement: Supplementary file 1 — Supplementary Material 1. [file 41598_2024_74837_MOESM1_ESM.docx]

**Supplemental Table 1.** Baseline Characteristics of Patients with SAE Categorized by 1-Year Survival Outcomes in the Training Set.

| **Characteristics** | **All patients (n=2751)** | **Survival patients (n=2194)** | **Nonsurvival patients (n=557)** | ***P-*value** |
| --- | --- | --- | --- | --- |
| Male, No. (%) | 1,671 (61%) | 1,372 (63%) | 299 (54%) | <0.001 |
| Age, median (IQR) | 67.40 (56.43, 76.44) | 66.17 (55.08, 75.42) | 72.64 (61.05, 81.13) | <0.001 |
| Height, median (IQR) (cm) | 170.00 (163.00, 178.00) | 170.00 (163.00, 178.00) | 168.00 (159.22, 175.00) | <0.001 |
| Weight, median (IQR) (kg) | 81.00 (68.90, 95.55) | 82.35 (70.30, 96.60) | 74.30 (62.20, 91.30) | <0.001 |
| **Race, No. (%)** | | | | |
| White | 1,925 (70%) | 1,540 (70%) | 385 (69%) | 0.622 |
| Hispanic | 74 (2.7%) | 63 (2.9%) | 11 (2.0%) | 0.243 |
| Black | 147 (5.3%) | 118 (5.4%) | 29 (5.2%) | 0.872 |
| Asian | 73 (2.7%) | 56 (2.6%) | 17 (3.1%) | 0.512 |
| Other | 532 (19%) | 417 (19%) | 115 (21%) | 0.381 |
| **Marital Status, No. (%)** | | | | |
| Married | 1,384 (50%) | 1,162 (53%) | 222 (40%) | <0.001 |
| Single | 673 (24%) | 538 (25%) | 135 (24%) | 0.889 |
| Widowed | 283 (10%) | 196 (8.9%) | 87 (16%) | <0.001 |
| Divorced | 184 (6.7%) | 141 (6.4%) | 43 (7.7%) | 0.275 |
| Other | 227 (8.3%) | 157 (7.2%) | 70 (13%) | <0.001 |
| **First Care Unit,** **No. (%)** | | | | |
| Medical ICU | 362 (13%) | 209 (9.5%) | 153 (27%) | <0.001 |
| Surgical ICU | 287 (10%) | 209 (9.5%) | 78 (14%) | 0.002 |
| Medical ICU/Surgical ICU | 307 (11%) | 177 (8.1%) | 130 (23%) | <0.001 |
| Neuro Surgical ICU | 13 (0.5%) | 8 (0.4%) | 5 (0.9%) | 0.155 |
| Trauma Surgical ICU | 281 (10%) | 222 (10%) | 59 (11%) | 0.742 |
| Other ICU | 1,501 (55%) | 1,369 (62%) | 132 (24%) | <0.001 |
| **Underlying Diseases,** **No. (%)** | | | | |
| Myocardial Infarction | 493 (18%) | 388 (18%) | 105 (19%) | 0.522 |
| Congestive Heart Failure | 709 (26%) | 509 (23%) | 200 (36%) | <0.001 |
| Chronic Pulmonary Disease | 729 (26%) | 517 (24%) | 212 (38%) | <0.001 |
| Diabetes Without Chronic Complication | 502 (18%) | 411 (19%) | 91 (16%) | 0.191 |
| Diabetes With Chronic Complication | 141 (5.1%) | 116 (5.3%) | 25 (4.5%) | 0.445 |
| Rheumatic Disease | 94 (3.4%) | 66 (3.0%) | 28 (5.0%) | 0.019 |
| Peptic Ulcer Disease | 78 (2.8%) | 39 (1.8%) | 39 (7.0%) | <0.001 |
| Peripheral Vascular Disease | 393 (14%) | 317 (14%) | 76 (14%) | 0.628 |
| Paraplegia | 40 (1.5%) | 31 (1.4%) | 9 (1.6%) | 0.721 |
| Metastatic Solid Tumor | 146 (5.3%) | 44 (2.0%) | 102 (18%) | <0.001 |
| Malignant Cancer | 282 (10%) | 139 (6.3%) | 143 (26%) | <0.001 |
| **CCI, median (IQR)** | 5.00 (3.00, 7.00) | 4.00 (3.00, 6.00) | 7.00 (5.00, 9.00) | <0.001 |
| **Vital Indicators, median (IQR)** | | | | |
| Heart Rate (beats/min) * | 70.00 (61.00, 80.00) | 69.00 (61.00, 78.00) | 74.00 (63.00, 86.00) | <0.001 |
| Heart Rate (beats/min) *** | 101.00 (90.00, 116.00) | 100.00 (89.00, 113.00) | 110.00 (96.00, 122.00) | <0.001 |
| Heart Rate (beats/min) ** | 84.00 (76.00, 95.00) | 83.00 (76.00, 93.00) | 90.00 (79.00, 102.00) | <0.001 |
| MAP (mmHg) * | 58.00 (52.00, 63.00) | 58.00 (53.00, 63.00) | 55.00 (48.00, 61.00) | <0.001 |
| MAP (mmHg) *** | 98.00 (89.00, 109.00) | 97.00 (89.00, 108.75) | 99.00 (87.00, 111.00) | 0.641 |
| MAP (mmHg) ** | 74.66 (70.10, 79.75) | 74.99 (70.73, 79.82) | 73.28 (67.89, 79.15) | <0.001 |
| Respiratory Rate (breaths/min) * | 12.00 (9.00, 14.00) | 11.00 (9.00, 14.00) | 13.00 (10.00, 15.50) | <0.001 |
| Respiratory Rate (breaths/min) *** | 27.00 (23.00, 31.00) | 26.00 (23.00, 30.00) | 28.00 (24.00, 33.00) | <0.001 |
| Respiratory Rate (breaths/min) ** | 18.06 (16.17, 20.65) | 17.78 (16.07, 20.07) | 19.71 (17.07, 22.81) | <0.001 |
| Temperature (°C) * | 36.33 (35.67, 36.61) | 36.33 (35.70, 36.67) | 36.33 (35.61, 36.56) | 0.118 |
| Temperature (°C) *** | 37.40 (37.00, 37.94) | 37.44 (37.06, 37.94) | 37.28 (36.94, 37.89) | <0.001 |
| Temperature (°C) ** | 36.83 (36.56, 37.17) | 36.84 (36.57, 37.18) | 36.79 (36.48, 37.13) | <0.001 |
| First day Urine Output (mL) | 1,710.00 (1,140.00, 2,475.00) | 1,822.50 (1,304.25, 2,555.00) | 1,150.00 (640.00, 1,815.00) | <0.001 |
| **Laboratory Indicators, median (IQR)** | | | | |
| Hemoglobin (g/L) * | 94.00 (82.00, 107.00) | 95.00 (83.00, 108.00) | 90.00 (80.00, 105.00) | <0.001 |
| Hemoglobin (g/L) *** | 113.00 (101.00, 126.00) | 114.00 (102.25, 127.00) | 107.00 (94.00, 121.00) | <0.001 |
| Platelets (K/uL) * | 145.00 (107.00, 200.00) | 143.50 (109.00, 193.00) | 152.00 (93.00, 235.00) | 0.264 |
| Platelets (K/uL) *** | 188.00 (144.00, 249.00) | 187.00 (146.00, 245.00) | 193.00 (129.00, 286.00) | 0.675 |
| White Blood Cells (K/uL) * | 9.90 (7.20, 13.00) | 9.90 (7.30, 12.80) | 10.10 (6.80, 14.10) | 0.254 |
| White Blood Cells (K/uL) *** | 14.50 (11.00, 19.10) | 14.65 (11.30, 18.90) | 14.10 (9.90, 19.80) | 0.044 |
| Anion Gap (mmol/L) * | 11.00 (10.00, 13.00) | 11.00 (10.00, 13.00) | 13.00 (11.00, 15.00) | <0.001 |
| Anion Gap (mmol/L) *** | 14.00 (12.00, 17.00) | 14.00 (12.00, 16.00) | 16.00 (14.00, 19.00) | <0.001 |
| Creatinine (μmmol/L) * | 70.72 (61.88, 97.24) | 70.72 (61.88, 88.40) | 88.40 (61.88, 150.28) | <0.001 |
| Creatinine (μmmol/L) *** | 88.40 (70.72, 123.76) | 79.56 (61.88, 106.08) | 114.92 (70.72, 185.64) | <0.001 |
| Glucose (mmol/L) * | 6.06 (5.28, 6.94) | 6.11 (5.39, 7.00) | 5.78 (4.83, 6.67) | <0.001 |
| Glucose (mmol/L) *** | 7.06 (6.06, 8.17) | 7.00 (6.00, 8.06) | 7.39 (6.28, 8.50) | <0.001 |
| Sodium (mEq/L) * | 137.00 (135.00, 139.00) | 137.00 (135.00, 139.00) | 137.00 (133.00, 140.00) | 0.007 |
| Sodium (mEq/L) *** | 140.00 (138.00, 142.00) | 140.00 (138.00, 142.00) | 139.00 (137.00, 142.00) | 0.007 |
| Potassium (mEq/L) * | 4.00 (3.60, 4.30) | 4.00 (3.70, 4.30) | 3.90 (3.50, 4.40) | 0.341 |
| Potassium (mEq/L) *** | 4.50 (4.20, 4.90) | 4.50 (4.20, 4.80) | 4.50 (4.10, 5.10) | 0.027 |
| Prothrombin Time (sec) * | 13.40 (12.30, 14.90) | 13.30 (12.20, 14.50) | 14.60 (12.80, 17.60) | <0.001 |
| Prothrombin Time (sec) *** | 15.30 (13.60, 17.50) | 15.20 (13.60, 17.10) | 16.30 (13.80, 21.50) | <0.001 |
| Partial Thromboplastin Time (sec) * | 28.80 (26.20, 33.00) | 28.40 (26.00, 31.90) | 31.50 (27.10, 39.10) | <0.001 |
| Partial Thromboplastin Time (sec) *** | 33.70 (29.00, 43.69) | 33.00 (28.80, 41.20) | 38.40 (30.80, 55.47) | <0.001 |
| Lactate (mmol/L) * | 1.30 (0.93, 1.70) | 1.20 (0.90, 1.60) | 1.40 (1.00, 2.00) | <0.001 |
| Lactate (mmol/L) *** | 2.20 (1.50, 3.11) | 2.20 (1.50, 3.10) | 1.80 (1.20, 3.20) | <0.001 |
| PH * | 7.32 (7.27, 7.37) | 7.32 (7.28, 7.37) | 7.32 (7.25, 7.40) | 0.255 |
| PH *** | 7.43 (7.39, 7.47) | 7.43 (7.40, 7.47) | 7.41 (7.36, 7.46) | <0.001 |
| PaCO2 (mmHg) * | 35.00 (32.00, 39.00) | 35.00 (32.00, 38.00) | 35.00 (30.00, 41.00) | 0.257 |
| PaCO2 (mmHg) *** | 46.00 (41.00, 52.00) | 47.00 (42.00, 52.00) | 44.00 (37.00, 52.00) | <0.001 |
| **Intervention Measures,** **No. (%)** | | | | |
| Vasoactive Agent Use | 1,695 (62%) | 1,375 (63%) | 320 (57%) | 0.024 |
| Albumin Use | 70 (2.5%) | 47 (2.1%) | 23 (4.1%) | 0.008 |
| Renal Replacement Therapy | 96 (3.5%) | 52 (2.4%) | 44 (7.9%) | <0.001 |
| Invasive Mechanical Ventilation | 1,684 (61%) | 1,348 (61%) | 336 (60%) | 0.629 |
| Supplemental Oxygen Therapy | 1,667 (61%) | 1,405 (64%) | 262 (47%) | <0.001 |
| **Disease severity Score, median (IQR)** | | | | |
| First day GCS * | 14.00 (9.00, 14.00) | 14.00 (10.00, 14.00) | 11.00 (6.00, 14.00) | <0.001 |
| First day SOFA | 6.00 (4.00, 9.00) | 6.00 (4.00, 8.00) | 8.00 (6.00, 12.00) | <0.001 |
| First day APS III | 46.00 (32.00, 69.00) | 41.00 (31.00, 61.00) | 68.00 (51.00, 90.00) | <0.001 |
| First day SAPS II | 37.00 (29.00, 48.00) | 36.00 (28.00, 45.00) | 46.00 (38.00, 56.00) | <0.001 |
| First day OASIS | 35.00 (29.00, 41.00) | 34.00 (28.00, 39.00) | 41.00 (34.00, 48.00) | <0.001 |
| First day LODS | 5.00 (3.00, 8.00) | 5.00 (3.00, 7.00) | 8.00 (5.00, 11.00) | <0.001 |
| **Outcomes** |  |  |  |  |
| ICU Mortality (%) | 238 (8.7%) | 0 (0%) | 238 (43%) | <0.001 |
| Hospital Mortality (%) | 279 (10%) | 0 (0%) | 279 (50%) | <0.001 |
| ICU LOS (days) | 3.13 (1.54, 6.04) | 2.71 (1.38, 5.04) | 5.35 (2.93, 9.99) | <0.001 |
| Hospital LOS (days) | 8.26 (5.41, 13.83) | 7.93 (5.30, 12.74) | 10.90 (6.63, 18.13) | <0.001 |

*: Minimum recorded values of indicators during the first 24 hours of ICU admission; **: Mean values of indicators during the first 24 hours of ICU admission; ***: Maximum recorded values of indicators during the first 24 hours of ICU admission. CCI, Charlson Comorbidity Index; ICU, Intensive Care Unit; IQR, Interquartile Range; GCS, Glasgow Coma Scale; APS III, Acute Physiology Score III; SOFA, Sequential Organ Failure Assessment; LODS, Logistic Organ Dysfunction System; MAP, Mean Arterial Pressure; PaCO2, Partial pressure of CO2; SAE, Sepsis-Associated Encephalopathy. SAPS II, Simplified Acute Physiology Score II; OASIS, Oxford Acute Severity of Illness Score; LOS, Length of Stay

**Supplemental Table 2.** Baseline Characteristics of Patients with SAE Categorized by 1-Year Survival Outcomes in the Validation Set.

| **Characteristics** | **All patients (n=1131)** | **Survival patients (n=902)** | **Nonsurvival patients (n=229)** | ***P-*value** |
| --- | --- | --- | --- | --- |
| Male, No. (%) | 667 (59%) | 538 (60%) | 129 (56%) | 0.363 |
| Age, median (IQR) | 66.90 (56.92, 76.39) | 66.12 (56.38, 75.19) | 71.02 (60.99, 79.66) | <0.001 |
| Height, median (IQR) (cm) | 170.00 (161.50, 178.00) | 170.00 (161.38, 178.00) | 168.00 (161.96, 177.05) | 0.135 |
| Weight, median (IQR) (kg) | 80.00 (67.10, 95.00) | 81.00 (68.00, 95.55) | 77.00 (63.60, 90.00) | 0.003 |
| **Race, No. (%)** | | | | |
| White | 780 (69%) | 633 (70%) | 147 (64%) | 0.080 |
| Hispanic | 37 (3.3%) | 33 (3.7%) | 4 (1.7%) | 0.146 |
| Black | 54 (4.8%) | 36 (4.0%) | 18 (7.9%) | 0.014 |
| Asian | 27 (2.4%) | 20 (2.2%) | 7 (3.1%) | 0.457 |
| Other | 233 (21%) | 180 (20%) | 53 (23%) | 0.287 |
| **Marital Status, No. (%)** | | | | |
| Married | 551 (49%) | 451 (50%) | 100 (44%) | 0.087 |
| Single | 268 (24%) | 210 (23%) | 58 (25%) | 0.516 |
| Widowed | 133 (12%) | 104 (12%) | 29 (13%) | 0.634 |
| Divorced | 85 (7.5%) | 69 (7.6%) | 16 (7.0%) | 0.734 |
| Other | 94 (8.3%) | 68 (7.5%) | 26 (11%) | 0.062 |
| **First Care Unit,** **No. (%)** | | | | |
| Medical ICU | 141 (12%) | 84 (9.3%) | 57 (25%) | <0.001 |
| Surgical ICU | 102 (9.0%) | 72 (8.0%) | 30 (13%) | 0.016 |
| Medical ICU/Surgical ICU | 140 (12%) | 74 (8.2%) | 66 (29%) | <0.001 |
| Neuro Surgical ICU | 5 (0.4%) | 4 (0.4%) | 1 (0.4%) | >0.999 |
| Trauma Surgical ICU | 120 (11%) | 96 (11%) | 24 (10%) | 0.943 |
| Other ICU | 623 (55%) | 572 (63%) | 51 (22%) | <0.001 |
| **Underlying Diseases,** **No. (%)** | | | | |
| Myocardial Infarction | 208 (18%) | 170 (19%) | 38 (17%) | 0.432 |
| Congestive Heart Failure | 316 (28%) | 239 (26%) | 77 (34%) | 0.032 |
| Chronic Pulmonary Disease | 320 (28%) | 243 (27%) | 77 (34%) | 0.045 |
| Diabetes Without Chronic Complication | 210 (19%) | 173 (19%) | 37 (16%) | 0.294 |
| Diabetes With Chronic Complication | 60 (5.3%) | 48 (5.3%) | 12 (5.2%) | 0.961 |
| Rheumatic Disease | 49 (4.3%) | 38 (4.2%) | 11 (4.8%) | 0.695 |
| Peptic Ulcer Disease | 21 (1.9%) | 15 (1.7%) | 6 (2.6%) | 0.407 |
| Peripheral Vascular Disease | 157 (14%) | 120 (13%) | 37 (16%) | 0.265 |
| Paraplegia | 14 (1.2%) | 11 (1.2%) | 3 (1.3%) | >0.999 |
| Metastatic Solid Tumor | 65 (5.7%) | 18 (2.0%) | 47 (21%) | <0.001 |
| Malignant Cancer | 134 (12%) | 53 (5.9%) | 81 (35%) | <0.001 |
| **CCI, median (IQR)** | 5.00 (3.00, 7.00) | 5.00 (3.00, 6.00) | 7.00 (5.00, 9.00) | <0.001 |
| **Vital Indicators, median (IQR)** | | | | |
| Heart Rate (beats/min) * | 70.00 (61.00, 79.00) | 69.00 (61.00, 78.00) | 74.00 (64.00, 85.00) | <0.001 |
| Heart Rate (beats/min) *** | 100.00 (90.00, 115.00) | 98.00 (89.00, 112.75) | 108.00 (95.00, 124.00) | <0.001 |
| Heart Rate (beats/min) ** | 84.00 (76.50, 95.00) | 83.00 (76.00, 93.00) | 88.00 (79.00, 101.00) | <0.001 |
| MAP (mmHg) * | 57.50 (52.00, 63.00) | 58.00 (53.00, 63.00) | 54.00 (49.00, 60.00) | <0.001 |
| MAP (mmHg) *** | 98.00 (89.00, 109.00) | 98.00 (89.00, 109.00) | 99.00 (88.00, 111.00) | 0.768 |
| MAP (mmHg) ** | 74.28 (69.62, 79.50) | 74.51 (70.19, 79.33) | 72.63 (67.30, 79.68) | 0.008 |
| Respiratory Rate (breaths/min) * | 12.00 (9.00, 14.00) | 12.00 (9.00, 14.00) | 12.00 (10.00, 15.00) | 0.003 |
| Respiratory Rate (breaths/min) *** | 27.00 (24.00, 31.00) | 26.00 (23.00, 31.00) | 28.00 (25.00, 34.00) | <0.001 |
| Respiratory Rate (breaths/min) ** | 18.23 (16.21, 20.71) | 17.98 (16.06, 20.44) | 19.25 (17.41, 22.32) | <0.001 |
| Temperature (°C) * | 36.33 (35.70, 36.61) | 36.33 (35.70, 36.61) | 36.33 (35.67, 36.56) | 0.649 |
| Temperature (°C) *** | 37.40 (37.00, 37.94) | 37.44 (37.00, 37.94) | 37.30 (36.94, 37.94) | 0.058 |
| Temperature (°C) ** | 36.83 (36.57, 37.18) | 36.85 (36.59, 37.18) | 36.73 (36.42, 37.19) | 0.006 |
| First day Urine Output (mL) | 1,765.00 (1,175.00, 2,445.50) | 1,855.00 (1,302.50, 2,518.75) | 1,228.00 (617.00, 2,160.00) | <0.001 |
| **Laboratory Indicators, median (IQR)** | | | | |
| Hemoglobin (g/L) * | 93.00 (82.00, 106.00) | 94.00 (82.00, 107.00) | 92.00 (81.00, 103.00) | 0.018 |
| Hemoglobin (g/L) *** | 112.00 (101.00, 126.00) | 114.00 (103.00, 127.00) | 105.00 (95.00, 120.00) | <0.001 |
| Platelets (K/uL) * | 147.00 (108.00, 206.00) | 143.00 (109.00, 197.00) | 172.00 (101.00, 257.00) | 0.006 |
| Platelets (K/uL) *** | 192.00 (148.00, 259.00) | 189.00 (149.00, 249.00) | 229.00 (137.00, 321.00) | 0.005 |
| White Blood Cells (K/uL) * | 10.00 (7.50, 12.90) | 9.90 (7.50, 12.70) | 10.70 (7.50, 15.00) | 0.036 |
| White Blood Cells (K/uL) *** | 15.10 (11.30, 18.95) | 15.10 (11.50, 18.88) | 15.20 (10.40, 19.70) | 0.565 |
| Anion Gap (mmol/L) * | 12.00 (10.00, 13.00) | 11.00 (10.00, 13.00) | 13.00 (11.00, 16.00) | <0.001 |
| Anion Gap (mmol/L) *** | 14.00 (12.00, 16.00) | 13.00 (12.00, 16.00) | 16.00 (14.00, 20.00) | <0.001 |
| Creatinine (μmmol/L) * | 70.72 (61.88, 97.24) | 70.72 (53.04, 88.40) | 97.24 (61.88, 167.96) | <0.001 |
| Creatinine (μmmol/L) *** | 88.40 (70.72, 123.76) | 88.40 (70.72, 114.92) | 123.76 (79.56, 203.32) | <0.001 |
| Glucose (mmol/L) * | 6.06 (5.22, 6.94) | 6.11 (5.28, 7.00) | 5.72 (4.89, 6.67) | <0.001 |
| Glucose (mmol/L) *** | 7.22 (6.11, 8.28) | 7.06 (6.06, 8.11) | 7.50 (6.61, 8.67) | <0.001 |
| Sodium (mEq/L) * | 137.00 (135.00, 139.00) | 137.00 (135.00, 139.00) | 137.00 (134.00, 140.00) | 0.373 |
| Sodium (mEq/L) *** | 140.00 (138.00, 142.00) | 140.00 (138.00, 142.00) | 140.00 (137.00, 142.00) | 0.133 |
| Potassium (mEq/L) * | 4.00 (3.60, 4.30) | 4.00 (3.60, 4.30) | 3.90 (3.60, 4.30) | 0.709 |
| Potassium (mEq/L) *** | 4.50 (4.20, 4.90) | 4.50 (4.20, 4.90) | 4.50 (4.10, 5.00) | 0.852 |
| Prothrombin Time (sec) * | 13.40 (12.30, 14.80) | 13.20 (12.20, 14.40) | 14.70 (12.90, 17.50) | <0.001 |
| Prothrombin Time (sec) *** | 15.30 (13.60, 17.50) | 15.15 (13.60, 17.10) | 16.00 (13.60, 20.40) | <0.001 |
| Partial Thromboplastin Time (sec) * | 29.00 (26.20, 33.25) | 28.55 (26.00, 32.20) | 32.30 (27.10, 37.70) | <0.001 |
| Partial Thromboplastin Time (sec) *** | 33.80 (29.35, 44.00) | 33.35 (29.23, 41.90) | 36.20 (30.50, 51.90) | <0.001 |
| Lactate (mmol/L) * | 1.30 (0.91, 1.70) | 1.22 (0.90, 1.65) | 1.40 (1.00, 2.20) | <0.001 |
| Lactate (mmol/L) *** | 2.20 (1.50, 3.20) | 2.29 (1.60, 3.10) | 2.02 (1.25, 3.50) | 0.199 |
| PH * | 7.33 (7.27, 7.37) | 7.32 (7.28, 7.37) | 7.34 (7.26, 7.40) | 0.273 |
| PH *** | 7.43 (7.39, 7.47) | 7.44 (7.40, 7.47) | 7.41 (7.36, 7.46) | <0.001 |
| PaCO2 (mmHg) * | 35.00 (31.00, 39.00) | 35.00 (31.00, 39.00) | 36.00 (31.00, 41.00) | 0.044 |
| PaCO2 (mmHg) *** | 46.00 (41.00, 52.00) | 46.50 (42.00, 52.00) | 45.00 (39.00, 52.00) | 0.003 |
| **Intervention Measures,** **No. (%)** | | | | |
| Vasoactive Agent Use | 725 (64%) | 598 (66%) | 127 (55%) | 0.002 |
| Albumin Use | 25 (2.2%) | 14 (1.6%) | 11 (4.8%) | 0.003 |
| Renal Replacement Therapy | 30 (2.7%) | 16 (1.8%) | 14 (6.1%) | <0.001 |
| Invasive Mechanical Ventilation | 696 (62%) | 559 (62%) | 137 (60%) | 0.551 |
| Supplemental Oxygen Therapy | 670 (59%) | 569 (63%) | 101 (44%) | <0.001 |
| **Disease severity Score, median (IQR)** | | | | |
| First day GCS * | 13.00 (8.00, 14.00) | 14.00 (9.00, 14.00) | 10.00 (6.00, 14.00) | <0.001 |
| First day SOFA | 6.00 (4.00, 9.00) | 6.00 (4.00, 8.00) | 8.00 (6.00, 12.00) | <0.001 |
| First day APS III | 46.00 (33.00, 70.00) | 42.00 (31.25, 62.00) | 65.00 (45.00, 95.00) | <0.001 |
| First day SAPS II | 38.00 (30.00, 49.00) | 37.00 (29.00, 46.00) | 46.00 (38.00, 57.00) | <0.001 |
| First day OASIS | 35.00 (29.00, 41.00) | 34.00 (29.00, 39.00) | 41.00 (34.00, 48.00) | <0.001 |
| First day LODS | 5.00 (3.00, 8.00) | 5.00 (3.00, 7.00) | 8.00 (5.00, 11.00) | <0.001 |
| **Outcomes** |  |  |  |  |
| ICU Mortality (%) | 82 (7.3%) | 0 (0%) | 82 (36%) | <0.001 |
| Hospital Mortality (%) | 106 (9.4%) | 0 (0%) | 106 (46%) | <0.001 |
| ICU LOS (days) | 3.23 (1.77, 6.24) | 2.95 (1.49, 5.43) | 5.11 (2.78, 9.73) | <0.001 |
| Hospital LOS (days) | 8.61 (5.42, 15.26) | 8.11 (5.38, 13.73) | 12.49 (5.91, 19.60) | <0.001 |

*: Minimum recorded values of indicators during the first 24 hours of ICU admission; **: Mean values of indicators during the first 24 hours of ICU admission; ***: Maximum recorded values of indicators during the first 24 hours of ICU admission. CCI, Charlson Comorbidity Index; ICU, Intensive Care Unit; IQR, Interquartile Range; GCS, Glasgow Coma Scale; APS III, Acute Physiology Score III; SOFA, Sequential Organ Failure Assessment; LODS, Logistic Organ Dysfunction System; MAP, Mean Arterial Pressure; PaCO2, Partial pressure of CO2; SAE, Sepsis-Associated Encephalopathy. SAPS II, Simplified Acute Physiology Score II; OASIS, Oxford Acute Severity of Illness Score; LOS, Length of Stay
